# Morphometry and Morphology of the Acromion Process and Its Implications in Subacromial Impingement Syndrome

**DOI:** 10.7759/cureus.44329

**Published:** 2023-08-29

**Authors:** Md Jawed Akhtar, Sanjay Kumar, Chandra Bhushan Chandan, Prabhat Kumar, Binod Kumar, Rajiv Ranjan Sinha, Avanish Kumar

**Affiliations:** 1 Department of Anatomy, Indira Gandhi Institute of Medical Sciences, Patna, IND; 2 Department of Physical Medicine and Rehabilitation, Nalanda Medical College Hospital, Patna, IND

**Keywords:** shoulder joint, subacromial impingement syndrome, morphometry, acromioplasty, scapula

## Abstract

Introduction

Subacromial impingement syndrome (SIS) is a common shoulder disorder characterized by pain and limited range of motion in the shoulder joint. It is frequently attributed to the compression or impingement of the rotator cuff tendons and bursa between the humeral head and the acromion process of the scapula during arm elevation. Subacromial impingement syndrome may arise as a result of the morphology of the acromion process, a bony protrusion at the top of the scapula that is important in the biomechanics of the shoulder joint. In order to detect potential anatomical differences that can predispose people to subacromial impingement syndrome, medical professionals and researchers need to have a thorough understanding of the morphometry and morphology of the acromion process.

Aims and objectives

The aim of the present study was to measure the morphometric and morphological characteristics of the acromion process in dried human scapulae that belonged to the North Indian population.

Materials and methods

This was a cross-sectional study that was carried out on 120 undamaged adult human scapula, of which 52 belonged to the right side and 68 belonged to the left side. Our study focused on analyzing the morphology of the acromion process as well as determining its maximum length, maximum breadth, acromio-coracoid distance, acromio-glenoid distance, and thickness. A statistical analysis of the observed parameters was carried out using the chi-square test and independent t-test with the help of Statistical Package for the Social Sciences (SPSS, IBM Corp., Armonk, NY) 24.0. Statistical significance was set at 0.05 (if the P-value ≤ 0.05, it is significant).

Results

We observed that the quadrangular shape (51.67%) of the acromion process was most commonly reported in our study, while the tubular (9.99%) shape was the least common. The difference in the incidences of various shapes of the acromion process on the right and left sides of the scapula was found to be statistically significant (p-value ≤ 0.05). In this study, the curved or type II acromion process was the most common type (53.34%) observed, while the least common shape reported was the hooked type (18.33%). The average length of the right acromion process was 44.52±6.61 mm, and the left acromion process was 45.13±6.35 mm. For the breadth, the right acromion had an average value of 28.31±4.67 mm, while the left had an average of 28.34±4.92 mm. The thickness of the right acromion measured 7.10±1.73 mm, and the left acromion was 7.53±1.44 mm. The acromio-coracoid distance on the right side was 34.59 ± 6.47 mm, and the left side was 37.46±6.22 mm. The acromio-glenoid distance was measured to be 32.31±5.87 mm on the right side and 33.18±5.39 mm on the left side.

Conclusions

Planning and carrying out an acromioplasty require an understanding of the morphometric parameters of the acromion process. Although there is a paucity of research on its morphometric evaluation in the North Indian population, the surgeons would be able to use these data as a reference.

## Introduction

Shoulder pain is a common musculoskeletal complaint that affects a significant proportion of the global population [1]. Among the various causes of shoulder pain, subacromial impingement syndrome (SIS) remains one of the most prevalent and clinically challenging conditions [1,2]. The acromion process and coracoacromial arch are pressed against by the rotator cuff tendons and subacromial bursa, which are structures that pass through the subacromial region, causing subacromial impingement syndrome. This condition is often associated with repetitive overhead activities, trauma, and degenerative changes, leading to inflammation, pain, and ultimately functional limitations. Over the years, researchers and clinicians have sought a comprehensive understanding of the anatomical factors contributing to the development of subacromial impingement syndrome. Among these factors, the morphology and morphometry of the acromion process have drawn significant attention. The acromion, a prominent bony projection of the scapula, plays a critical role in shoulder biomechanics, serving as an attachment site for several ligaments and muscles involved in shoulder movements and stability. The coracoid process, coraco-acromial ligament, and acromion process combine to produce the coraco-acromial arch [3]. This arch is a structure that is largely non-elastic. The long head of the biceps brachii passes closely beneath the coracoacromial arch and is traversed by the subacromial bursa and rotator cuff tendons [4]. Mechanical impingement results from a narrowing of the available space due to either acquired or congenital factors. Understanding the morphometry of the acromion process is important because its improper presentation may cause shoulder impingement syndrome. Thus, the shape and dimensions of the acromion have been linked to variations in the subacromial space, potentially influencing the occurrence and severity of impingement. The relationship between the acromion process and subacromial impingement syndrome has been the subject of extensive investigation in recent years. Despite numerous studies, the exact association between acromion morphology, morphometry, and SIS remains complex and multifactorial. However, advancements in imaging techniques, such as computed tomography (CT) scans, magnetic resonance imaging (MRI), and three-dimensional (3D) reconstruction, have provided researchers with valuable tools to analyze and quantify the acromion's structural features more accurately. This research aims to explore the intricate relationship between the morphometry and morphology of the acromion process and its implications for subacromial impingement syndrome. This study aims to contribute to the limited research on the morphometric analysis of the acromion process in the North Indian population.

Aims and objectives

The goal of the current study was to quantify the morphometric and morphological features of the acromion process of dried human scapulae (right and left sides) that belonged to the North Indian population.

## Materials and methods

This study was a cross-sectional study that was carried out on 120 undamaged dried adult human scapula, of which 52 belonged to the right side and 68 belonged to the left side. They were collected from the Department of Anatomy, Indira Gandhi Institute of Medical Sciences, Patna, Bihar (India). The age and gender of the bones used in the study were unknown. This study was of one-year duration and was conducted after approval of the Institute Ethical Committee of the Indira Gandhi Institute of Medical Sciences, Patna, Bihar (India), with letter number 648/IEC/IGIMS/2022. Only those well-preserved scapulae were included in the study that had an intact, complete acromion process, were suitable for accurate morphological observations, and belonged to adult individuals. Bones that were extensively damaged, fragmented, healed fractures, or showed significant deformities affecting the acromion process of the scapulae were excluded from the study.

Linear measurements were taken with the help of a digital vernier caliper, which had a sensitivity of 0.01 mm, and the least count observed was 0.01 mm. Measurements were taken twice, and the average was included in the analysis. The following parameters were studied.

Morphology of the acromion process

The incidences of various shapes of the acromion, i.e., triangular, quadrangular, or tubular, were noted. A triangular acromion was identified by its shape, which resembles a triangle. It has a relatively broad base that attaches to the spine of the scapula and a pointed tip that extends toward the lateral aspect of the shoulder joint. While a quadrangular acromion had a shape that resembled a quadrilateral or rectangle and typically had a more square or rectangular appearance compared to the other shapes, a tubular acromion was identified by its elongated and cylindrical shape, which had a more consistent width along its length. The three forms of acromion process morphology, type I (flat), type II (curved), and type III (hooked), were also examined from the lateral side of the scapula, and their occurrences were noted. In accordance with Bigliani et al. [5], i.e., type I acromion was characterized by a flat and relatively straight shape without notable downward or forward curvature. Type II (curved) had a slight curvature downward and forward, and the undersurface angle was moderately curved, creating a gentle slope. A type III acromion (hooked) included those that were hooked or curved more significantly than a type II acromion, and the undersurface angle was steeply curved, creating a pronounced hook shape.

Maximum length of the acromion process

**Figure 1:**
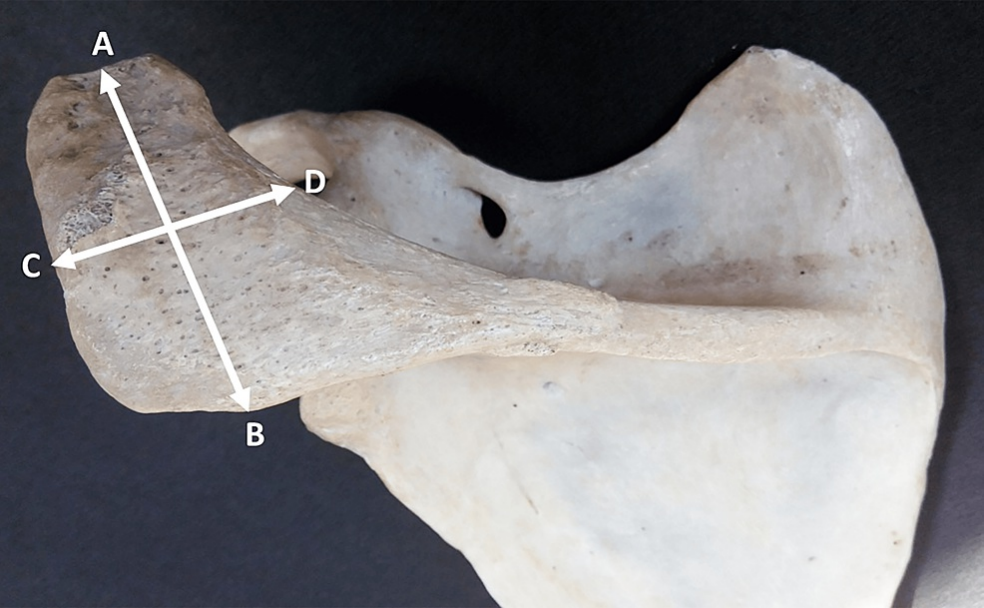
Showing different points used for the measurement of length and breadth of the acromion process.

The distance from the tip of the acromion process and the midpoint of its posterior border (from point A to point B) (Figure 1). Pearson correlation coefficients between the two observers were 1.00 (p-value < 0.001) for both the right and left sides.

Maximum breadth of the acromion process

The distance between the lateral border of the acromion process and its medial border at the midpoint (from point C to point D) (Figure 1). Pearson correlation coefficients between the two observers were 1.00 (p-value < 0.001) for both the right and left sides.

Acromio-coracoid distance

**Figure 2:**
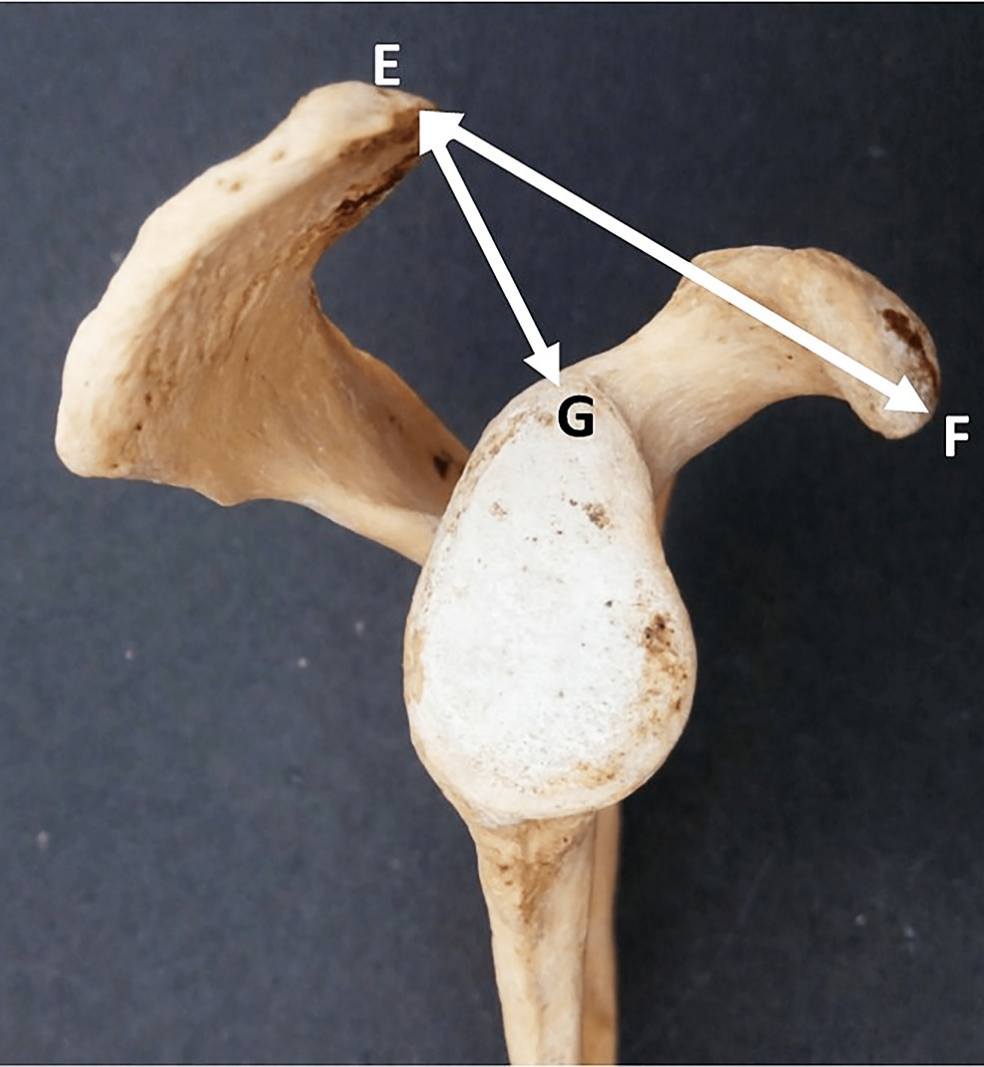
Showing different points used for the measurement of acromio-coracoid distance and acromio-glenoid distance.

The distance between the tips of the acromion process and the coracoid process (from point E to point F) (Figure 2). Pearson correlation coefficients between the two observers were 1.00 (p-value < 0.001) for both the right and left sides.

Acromio-glenoid distance

The distance from the tip of the acromion process to the supraglenoid tubercle (from point E to point G) (Figure 2). Pearson correlation coefficients between the two observers were 1.00 (p-value < 0.001) for both the right and left sides.

Thickness of the acromion process

**Figure 3:**
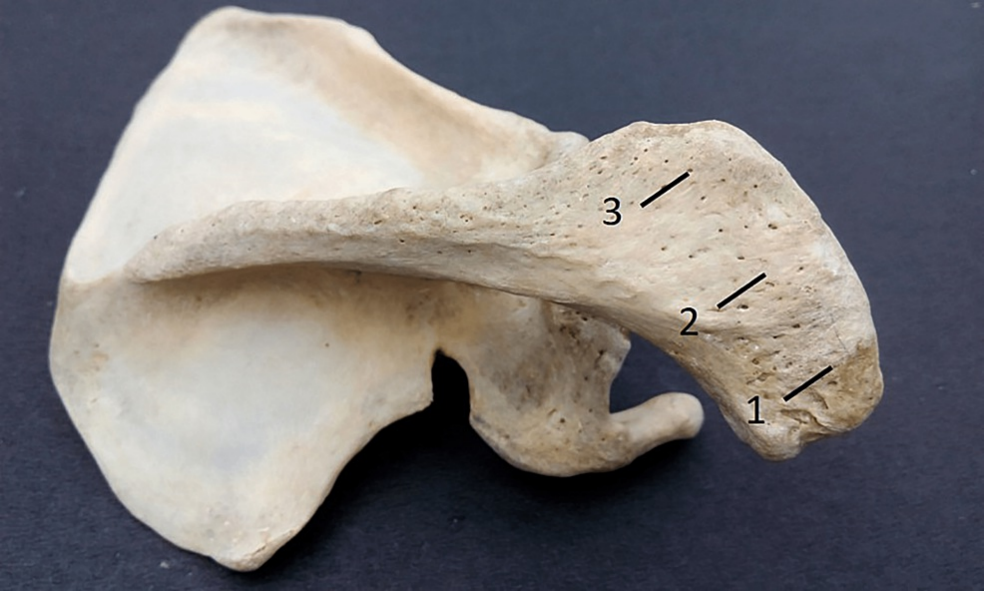
Showing different points used for the measurement of the thickness of the acromion process (at points 1, 2, and 3).

At three separate locations along the lateral border, this was measured. The anterior end of the acromion process was the first location, the centre of the acromion process was the second, and the posterior end was near the third (Figure 3). The average of these three readings was then used to calculate the acromion process' thickness. Pearson correlation coefficients between the two observers were 1.00 (p-value < 0.001) for both the right and left sides.

Statistical analysis

Continuous variables were expressed in terms of mean and standard deviation, while categorical variables were expressed in terms of percentage. The Pearson correlation coefficient was used to evaluate the interobserver agreement, and it was calculated based on two sets of data obtained by two observers at different times. Statistical analysis of the observed parameters was carried out using the chi-square test and independent t-test with the help of Statistical Package for the Social Sciences (SPSS, IBM Corp., Armonk, NY) 24.0. Statistical significance was set at 0.05 (if the P-value ≤ 0.05, it is significant).

## Results

**Figure 4:**
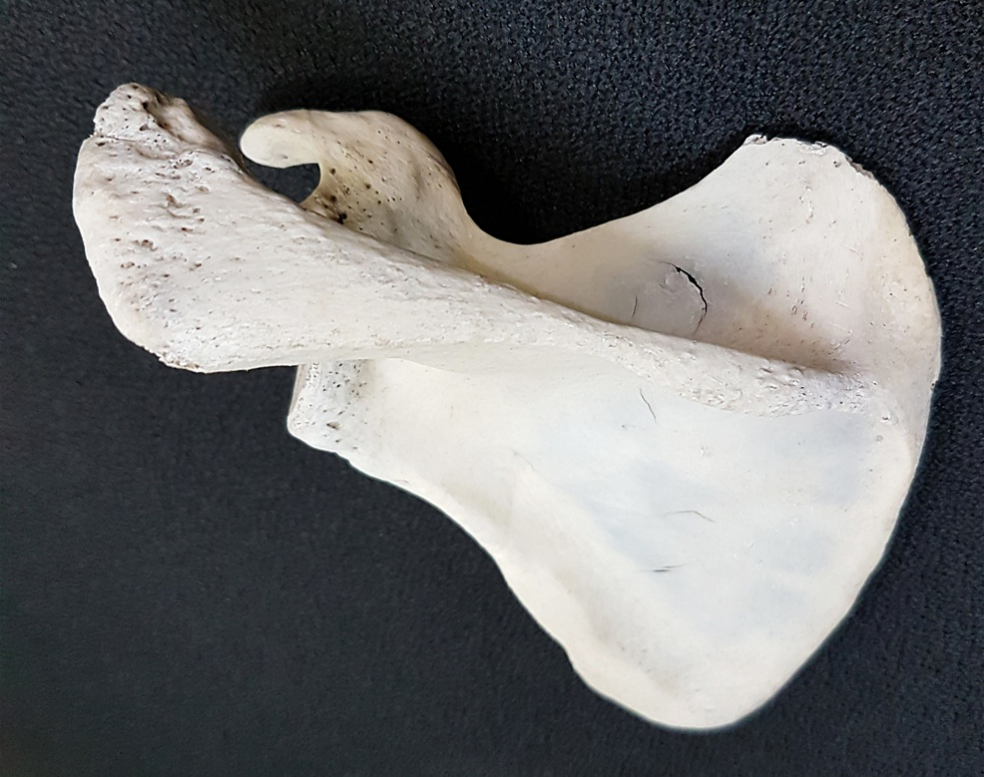
Scapula having acromion process of triangular shape.

**Figure 5:**
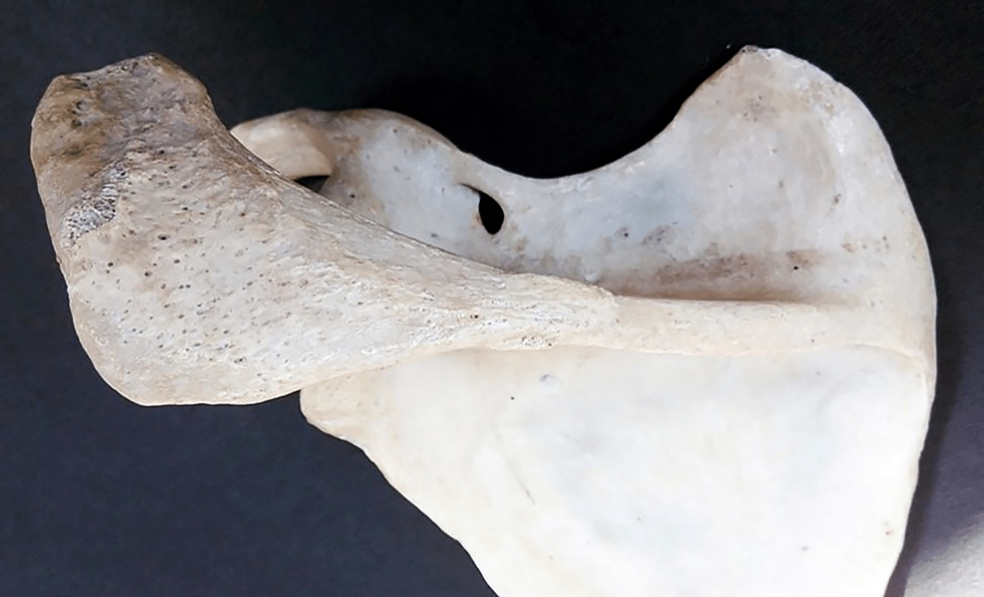
Scapula having acromion process of quadrangular shape.

**Figure 6:**
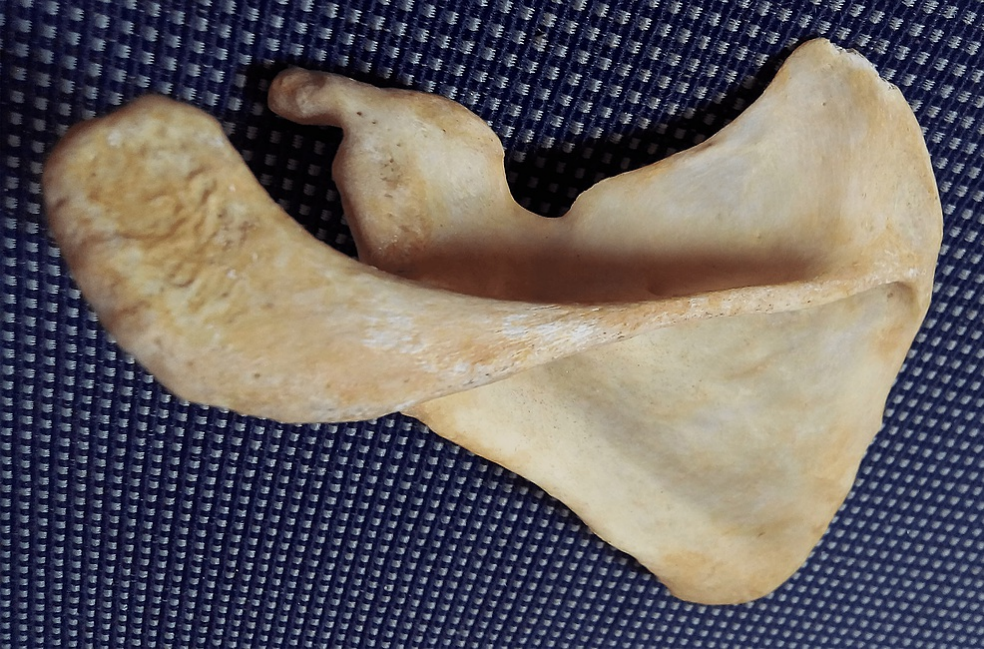
Scapula having acromion process of tubular shape.

The present study found that the most frequent shape of the acromion process was triangular (21.67%) on the right side and quadrangular (36.67%) on the left side. While overall quadrangular shape (51.67%) was most commonly reported, the second commonest shape was quadrangular (15%) on the right side and triangular (16.67%) on the left side, while overall triangular shape (38.34%) was next to quadrangular (51.67%). Overall, the least common shape recorded was tubular (9.99%); this was also the least common on the right (6.66%) as well as left (3.33%) sides (Table 1 and Figures 4-6). The difference in the incidences of various shapes of the acromion process on the right and left sides of the scapula was found to be statistically significant (p-value ≤ 0.05).

**Table 1:** Comparisons of occurrences of different shapes of the acromion process on the right and left side.

Shape	Right (n=52)	Left (n=68)	Total (n=120)	Chi-square X^2^	P-value
1. Triangular	26 (21.67%)	20 (16.67%)	46 (38.34%)	11.082	0.003
2. Quadrangular	18 (15%)	44 (36.67%)	62 (51.67%)		
3. Tubular	8 (6.66%)	4 (3.33%)	12 (9.99%)		
Total	52 (43.33%)	68 (56.67%)	120 (100%)		

**Figure 7:**
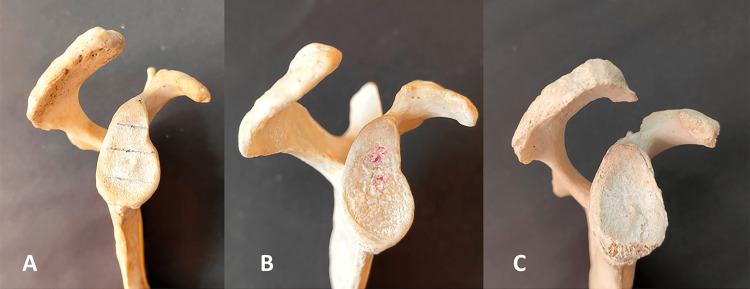
Different types of the acromion process of right-sided scapula. (A) Flat type (type I), (B) curved type (type II), and (C) hooked type (type III).

**Figure 8:**
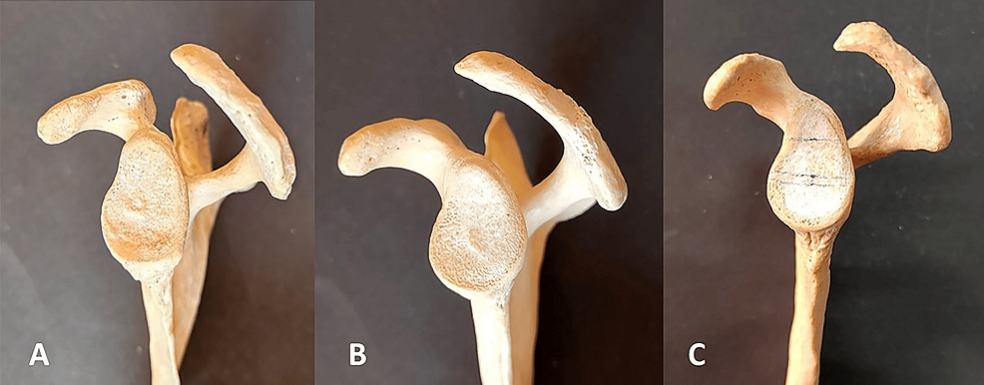
Different types of the acromion process of left-sided scapula. (A) Flat type (type I), (B) curved type (type II), and (C) hooked type (type III).

According to the classification by Bigliani et al. [5], we categorized the acromial morphology into the following three types (Table 2 and Figures 7-8).

**Table 2:** Showing occurrences of different types of the acromion process.

Types of acromion process	Right (n=52)	Left (n=68)	Total (n=120)	Chi-square X^2^	P-value
1. Type I: flat	16 (13.33%)	18 (15%)	34 (28.33%)	0.426	0.809
2. Type II: curved	26 (21.67%)	38 (31.67%)	64 (53.34%)		
3. Type III: hooked	10 (8.33%)	12 (10%)	22 (18.33%)		
Total	52 (43.33%)	68 (56.67%)	120 (100%)		

In this study, the curved or type II acromion process was the most frequently observed (53.34%) on both the left (31.67%) and right (21.67%) sides. The second most common type reported was the flat type (type I) acromion process on the right (13.33%) and the left side (15%). The overall incidence of flat type (type I) was 28.33%. While the least common shape reported in the present study was the hooked type, i.e., type III, both on the right (8.33%) as well as left (10%) sides, the overall incidence was 18.33% (Table 2 and Figures 7-8). The difference in the incidences of various types of the acromion process on the right and left sides of the scapula was not statistically significant (p-value ≥ 0.05).

Based on observations, the average length of the right acromion process was 44.52±6.61 mm, with a range of 34.10 mm to 54.20 mm. The left acromion process had an average length of 45.13±6.35 mm, with a range of 33.20 mm to 56.1 mm. As for the breadth, the right acromion had an average value of 28.31±4.67 mm (range of 20.20 mm to 35.20 mm), while the left had an average of 28.34±4.92 mm (range of 18.7 mm to 36.2 mm). There were no statistically significant differences in length or breadth between the right and left acromion processes (Table 3).

**Table 3:** Showing different dimensions of the acromion process. All measurements were taken in mm.

Sr. No.	Parameters	Right side		Left side		P-value	t-value
		Range (min-max)	Mean ± SD	Range (min-max)	Mean ± SD		
1	Length of the acromion process	34.10–54.20	44.52±6.61	33.20–56.1	45.13±6.35	0.611	−0.510
2	Breadth of the acromion process	20.20–35.20	28.31±4.67	18.7–36.2	28.34±4.92	0.968	−0.040
3	Thickness of the acromion process	4.10–9.98	7.10±1.73	4.60–11.24	7.53±1.44	0.141	−1.481
4	Acromio-coracoid distance	25.86–47.25	34.59±6.47	27.87–50.18	37.46±6.22	0.015	−2.458
5	Acromio-glenoid distance	22.15–41.22	32.31±5.87	22.68–42.84	33.18±5.39	0.258	−1.137

The study found that the thickness of the right acromion measured between 4.10 mm and 9.98 mm, with an average of 7.10±1.73 mm. The left acromion measured between 4.60mm and 11.24 mm, with an average of 7.53±1.44 mm. The difference in thickness between the right and left acromion processes was not statistically significant, as shown in Table 3.

The distance between the acromion and coracoid bones was measured on both the right and left sides. The range of the distances on the right side was between 25.86 and 47.25 mm, with a mean value of 34.59±6.47 mm. On the left side, the range was from 27.87 to 50.18 mm, with a mean value of 37.46±6.22 mm. Statistical analysis showed that there were significant differences between the distances on the right and left sides (Table 3).

The average acromion-glenoid distance was measured to be 32.31±5.87 mm (range 22.15 to 41.22 mm) on the right side and 33.18±5.39 mm (range 22.68 to 42.84 mm) on the left side. However, this difference was not statistically significant, as shown in Table 3.

## Discussion

On the morphology of the acromion process of the scapulae, numerous studies have been done [6-8]. Despite numerous studies, the exact association between acromion morphology, morphometry, and SIS remains complex and multifactorial. There may be a correlation between acromial morphology, shoulder impingement, and rotator cuff tears [1,2].

Mansur et al. [6] reported three different shapes of the acromion process in their study, among which the quadrangular shape (52.94%) was the most common. We also found that the quadrangular shape (51.67%) was the most common shape in the present study. Similar findings were also reported by other authors [7,8]. The incidence of these three different shapes of the acromion in our study is compared with the studies of different authors in Table 4.

**Table 4:** Comparison of incidence of different shapes of the acromion process of the scapula.

Sr. No.	Authors (Year)	Study Population	Triangular (%)	Quadrangular (%)	Tubular (%)
1.	Mansur et al. [6]	Nepalese	36.76%	52.94%	10.29%
2.	Gupta et al. [7]	South Indian	8%	44%	---
3.	Sinha et al. [8]	Indian	31.14%	55.73%	13.11%
4.	Present study	North Indian	38.34%	51.67%	9.99%

Bigliani et al. [5] documented three primary types of acromial shapes, among which the flat type was type I, the curved type was type II, and the hooked type was type III. The relative percentages for these three different types of acromion processes were as follows: 8.6% for type I, 42.0% for type II, and 38.6% for type III. They found that type III acromia was most closely related to rotator cuff injuries. We observed that the incidences of these three types were 28.33%, 55.34%, and 18.33%, respectively. In Table 5, we compare the occurrence of these three distinct acromion types in our study with those found in previous studies by various authors.

**Table 5:** Comparison of incidence of different types of the acromion process of the scapula.

Sr. No.	Authors (year)	Study population	Type I (%)	Type II (%)	Type III (%)
1.	Banas et al. [9]	American	39%	51%	10%
2.	Getz et al. [10]	Greek	22.8%	68.5%	8.6%
3.	Nicholson et al. [11]	North American	32%	42%	26%
4.	Coskun et al. [12]	Turkish	10%	73%	17%
5.	Nastsis et al. [13]	German	12.1%	56.5%	28.8%
6.	Sangiampong et al. [14]	Thai	3.2%	93.5%	3.2%
7.	Paraskevas et al. [4]	Greek	26.1%	55.6%	18.1%
8.	Tangtrakulwanich and Kapkird [15]	Thai	84.5%	10.7%	4.8%
9.	Singh et al. [3]	West Indian	22.5%	38.8%	38.8%
10.	Schetino et al. [16]	Brazilian	5.6%	57.89%	36.84%
11.	Gupta et al. [7]	South Indian	32%	22%	46%
12.	El Din and Ali [17]	Egyptian	26.9%	45.62%	15%
13.	Naidoo et al. [18]	African	34.6%	51.1%	14%
14.	Vinay and Sivan [19]	South Indian	37.1%	47.5%	15.2%
15.	Panigrahi and Mishra [20]	Indian	25.59%	56.90%	17.51%
16.	Guo et al. [21]	Chinese	47.3%	50%	3%
17.	Sinha et al. [8]	Indian	24.59%	49.18%	26.22%
18.	Alraddadi et al. [22]	English	2%	55%	43%
19.	Prasad et al. [23]	South Indian	57.14%	40%	2.85%
20.	Chaimongkhol et al. [24]	Thai	9.2%	78.8%	12%
21.	Vinay et al. [25]	South Indian	9%	49%	42%
22.	Koca et al. [26]	Turkish	21%	62%	17%
23.	Present study	North Indian	28.33%	53.34%	18.33%

We observed that type II was the most common and type III was the least commonly reported in our study. A similar observation was also reported in many other studies [4,9-11,17-21]. While some authors found that type II was the most common and type I was the least common in their study [3,8,12,13,16,21,22,24,25].

Sangiampong et al. [14] found that the most common type was type II, while types I and III were equally present in their study. Tangtrakulwanich and Kapkird [15] reported that type I was the most common and type III was the least common in their study. While Gupta et al. [7] observed that type III was the most common and type II was the least common in their observation, Prasad et al. [23] reported that type I was the most common and type II was the least common. There could be a variation in the results due to differences in ethnicity and the chosen research methodology. According to Bigliani et al. [5], hooked acromion is most often linked to rotator cuff injuries and subacromial impingement syndrome.

Research conducted by Tangtrakulwanich and Kapkird [15] identified four independent risk factors for impingement syndrome: smoking, occupation, acromion shape, and sleeping position. Apart from the lateral acromion, the anterior edge and undersurface of the anterior part of the acromion, the coracoacromial ligament, and the acromioclavicular joint are possible sites for impingement [27]. The posterior part of the acromion is not involved in the impingement process. Therefore, it is thought that lateral acromionectomy or total acromionectomy, which involves removing the lateral edge without detaching the deltoid, unnecessarily weakens the deltoid [28]. This issue led to the recommendation of a procedure known as an anterior acromioplasty, which entails the removal of the coracoacromial ligament as well as the anterior edge and undersurface of the anterior-most area of the acromion process [27,28]. Anterior acromioplasty has taken the place of these methods for removing the rotator cuff [28]. The thickness of the acromion is an important consideration for treating subacromial impingement syndrome surgically with acromioplasty, which entails removing the anterior section of the acromion [24]. The importance of the acromial characteristics assessed in the current study is significant in light of the fact that acromioplasty is currently the recommended procedure for treating subacromial impingement syndrome.

The acromial length in the present study was 44.52±6.61 mm on the right side and 45.13±6.35 mm on the left side, which is closer to the observations of Paraskevas et al. [4], Mansur et al. [6], Singh et al. [3], Chaimongkhol et al. [24] and Vinay et al. [25]. The acromial breadth in the present study was 28.31±4.67 mm on the right side and 28.34±4.92 mm on the left side, which is closer to the observations of Vinay and Sivan [19] and Vinay et al. [25].

The thickness of the acromion process in the present study was 7.10±1.73 mm on the right side and 7.53±1.44 mm on the left side, which is closer to the observations of Gupta et al. [7], Sinha et al. [8], and Priya and Jain [29].

The acromio-coracoid distance in the present study was 34.59±6.47 mm on the right side and 37.46±6.22 mm on the left side, which is closer to the observations of Singh et al. [3], Sinha et al. [8], Vinay and Sivan [19], Panigrahi and Mishra [20], and Priya and Jain [29]. Since the coracoacromial distance represents the length of the coracoacromial ligament, it can be helpful when doing the coracoacromial ligament excision, which significantly reduces discomfort in patients with impingement [30].

The acromio-glenoid distance in the present study was 32.31±5.87 mm on the right side and 33.18±5.39 mm on the left side, which is closer to the observations of Mansur et al. [6]. When the coracoacromial arch is more than 15 mm away from the supraglenoid tubercle, the acromion process begins to degenerate [29].

In Table 6, we compared the different morphometric parameters of the acromion process found in previous studies by various authors.

**Table 6:** Comparison of the mean value of different parameters of the acromion process of the scapula.

Sr. No.	Authors (year)	Study population		Acromial length (mm)	Acromial breadth (mm)	Acromial thickness (mm)	Coraco-acromial distance (mm)	Acromio-glenoid distance (mm)
1.	Paraskevas et al. [4]	Greek		46.10	22.30	8.80	28.10	17.70
2.	Mansur et al. [6]	Nepalese	Right	46.46	26.63	-	26.63	31.00
			Left	45.57	27.23	-	39.39	31.97
3.	Singh et al. [3]	West Indian		46.10	23.20	6.60	37.50	27.00
4.	Gupta et al. [7]	South Indian	Right	41.60	23.20	7.30	31.80	25.30
			Left	42.50	24.90	7.40	30.30	24.30
5.	El Din and Ali [17]	Egyptian		52.81	32.05	9.06	31.34	27.39
6.	Vinay and Sivan [19]	South Indian	Right	42.48	26.68	-	33.81	29.79
			Left	42.46	26.44	-	34.34	30.36
7.	Panigrahi and Mishra [20]	Indian	Right	41.72	21.42	6.68	37.49	26.39
			Left	38.97	21.57	6.59	37.23	24.20
8.	Sinha et al. [8]	Indian		41.23	22.12	7.01	35.94	28.28
9.	Prasad et al. [23]	South Indian		-	-	-	30.90	24.90
10.	Chaimongkhol et al. [24]	Thai		43.55	24.52	8.53	29.72	25.96
11.	Vinay et al. [25]	South Indian	Right	48.76	27.43	-	38.26	27.62
			Left	45.50	26.67	-	37.17	27.14
12.	Priya and Jain [29]	North Indian		41.65	24.50	7.62	33.35	29.41
13.	Koca et al. [26]	Turkish		36.21	-	-	30.48	-
14.	Present study	North Indian	Right	44.52	28.31	7.10	34.59	32.31
			Left	45.13	28.34	7.53	37.46	33.18

Limitations

Limitations of this study is that the age and sex of the scapulae were not studied as it was not available.

## Conclusions

Planning and carrying out an acromioplasty require an understanding of the morphometric parameters of the acromion process. Although there is a paucity of research on its morphometric evaluation in the North Indian population, the surgeons would be able to use these data as a reference. Additionally, knowing the acromion's original thickness greatly aids orthopaedic surgeons in determining how much bone needs to be shaved and how much can be preserved to maintain the rotator cuff's dynamics after surgery. The measurements of additional factors let the surgeon decide on the best strategy and precise operating method.
